# Hantaviruses: Current insights, lessons from the *MV Hondius* outbreak, and an urgent call to action

**DOI:** 10.1371/journal.ppat.1014756

**Published:** 2026-11-13

**Authors:** Nikhil Chakravarty, Vrithika Vaithi, Arjit Vijey Jeyachandran, Rakshiah Arunachalam, Anne Zaiss, Anna Bonazzi, Mark Parcells, Gustavo Garcia, Arunachalam Ramaiah, Vaithilingaraja Arumugaswami

**Affiliations:** 1 Department of Medical Education, California University of Science and Medicine, Colton, California, United States of America; 2 Department of Molecular and Medical Pharmacology, University of California, Los Angeles, California, United States of America; 3 Department of Animal and Food Sciences, University of Delaware, Newark, Delaware, United States of America; 4 Georgia Public Health Laboratory, Georgia Department of Public Health, Decatur, Georgia, United States of America; 5 California NanoSystems Institute, University of California, Los Angeles, California, United States of America; 6 Department of Bioengineering, Samueli School of Engineering, University of California, Los Angeles, California, United States of America; 7 Eli & Edythe Broad Center of Regenerative Medicine and Stem Cell Research, University of California, Los Angeles, California, United States of America; University of Iowa, UNITED STATES OF AMERICA

## Abstract

In May 2026, a cluster of laboratory-confirmed Andes virus (ANDV) infections was reported among passengers aboard the *MV Hondius, a* cruise ship that originated in Argentina and sailed through the South Atlantic. The exposed population included individuals from 23 countries and resulted in at least 13 cases, including three deaths, representing the first ANDV outbreak with a multi-continental range and providing further evidence consistent with human-to-human transmission of ANDV. There are currently no approved treatments or vaccines for ANDV, necessitating rapid development of drug and prophylactic pipelines. The outbreak highlighted the strengths and inherent weaknesses of our existing public health preparedness infrastructure in response to emerging infectious diseases. In this Pearl, we highlight the epidemiology, molecular virology, evolution, virus-host interactions, clinical management, therapeutic development, and public health response to ANDV. We emphasize the need to expand genomic surveillance, standardize diagnostic protocols across public health laboratories, and accelerate the clinical translation of promising antiviral candidates to strengthen pandemic preparedness infrastructure for understudied and emerging infectious diseases.

## A primer on the *MV Hondius* outbreak

Andes virus (ANDV) is the only hantavirus capable of human-to-human transmission, underscoring its potential for outbreaks that extend beyond the traditional rodent-to-human route. This significance became particularly clear in May 2026, when the World Health Organization (WHO) was notified of several cases of severe respiratory illness aboard the *MV Hondius, a* cruise ship carrying 147 passengers and crew from 23 countries, as it transited the South Atlantic from Argentina. Only a few days later, at least 13 cases, including three deaths, were identified and/or laboratory-confirmed, including secondary cases among contacts of the index patient [1], as well as plane passengers in close proximity to confirmed cases on a flight to South Africa [2] and a repatriation flight from Tenerife [1–6]. Contact tracing and quarantine measures were coordinated through the WHO National IHR (International Health Regulations) Focal Points and implemented across several countries, including the United Kingdom, South Africa, and the United States [2,3,7–10].

However, this outbreak is not the only recent notable case of hantaviral spread. In 2025, Betsy Arakawa, the wife of renowned actor Gene Hackman, passed away due to hantavirus infection in New Mexico, as did three people in Mammoth Lakes, California [11,12]. Hantavirus outbreaks highlight the challenge posed to public health agencies by neglected zoonotic pathogens—especially those that are ecologically complex, geographically distributed, understudied, and underfunded, yet capable of causing lethal outbreaks with little to no warning, thereby creating major public health concerns. As such, we aim to synthesize a brief primer on our current knowledge of hantaviruses and to propose areas for further research and development of emergency preparedness and capacity building within existing public health infrastructure.

## What we know

### Epidemiologic history and global burden of Hantaviruses

Old World (e.g., Hantaan [HTNV], Puumala [PUUV], Dobrava, and Seoul [SEOV] virus) and New World (e.g., Sin Nombre virus [SNV], ANDV) hantaviruses are primarily carried and spread asymptomatically in rodent reservoirs. The history of hantaviruses can be traced back to the Korean War in the 1950s, when over 3,000 troops stationed along the Hantaan River fell ill with a previously unknown virus, now known as HTNV [13] (Fig 1). The first recorded outbreak of hantaviruses in the United States occurred in 1993 in the Four Corners region of Arizona, Colorado, New Mexico, and Utah [14,15]. Patients passed away from fever, myalgias, thrombocytopenia, and pulmonary edema with hypotensive shock, and the Centers for Disease Control and Prevention (CDC) established the presence of SNV and its deer mouse reservoir. The spread of SNV was later associated with the 1991–1992 El Niño event, which caused warmer winters and increased spring rainfall, inciting a boom in the rodent population and promoting viral spread [14,15]. ANDV was first detected during a small outbreak in the Patagonia region of southern Argentina in 1995, with the first major outbreak of ANDV occurring in 2018–2019 in Epuyén, Argentina, where three distinct superspreader events occurred with no relation to inciting exposure to a rodent reservoir, confirming its ability to spread between humans [16].

**Fig 1:**
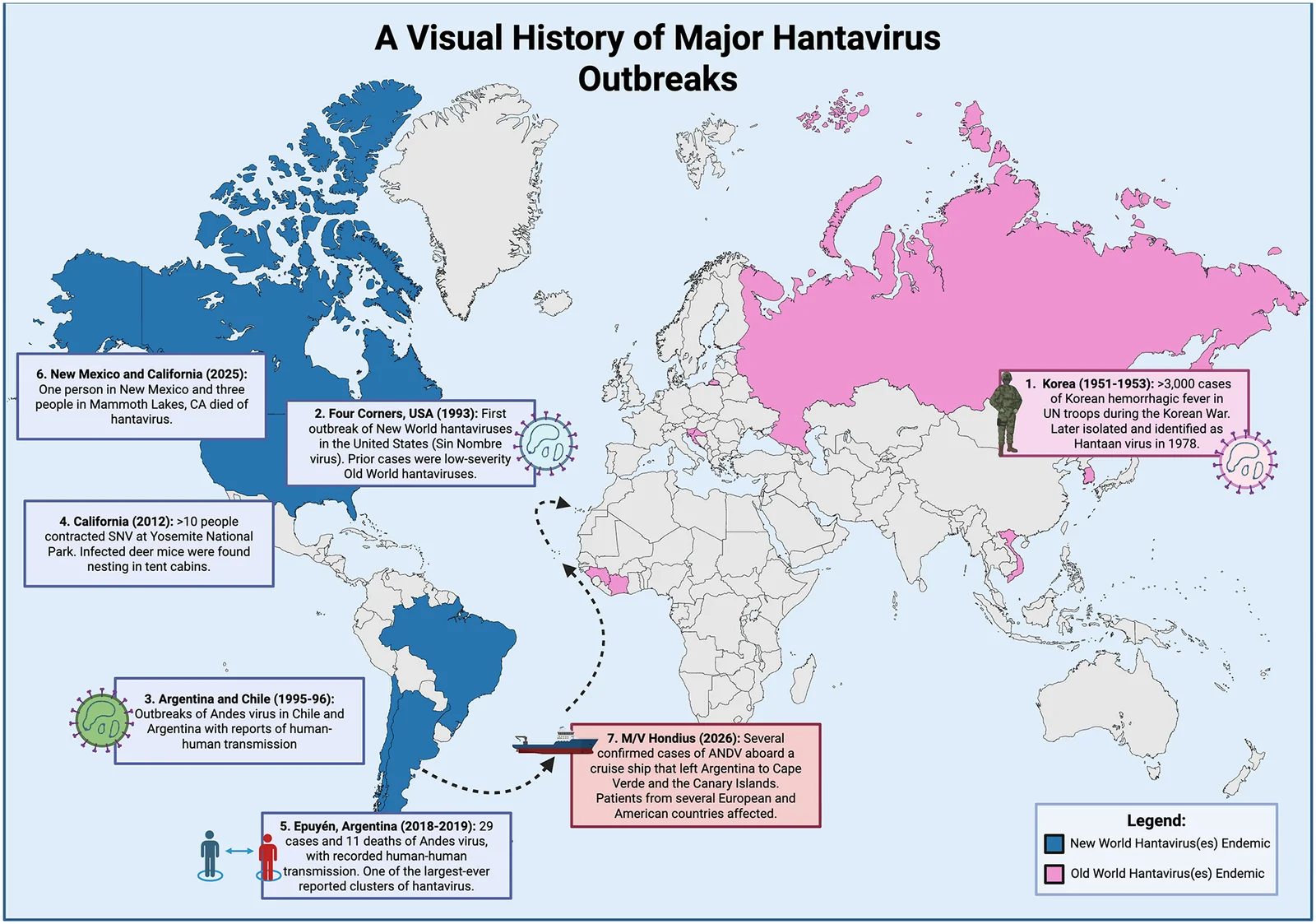
A visual history of major Hantavirus outbreaks. A schematic showing all major hantavirus outbreaks to date. Descriptive boxes are color-coded according to the type of hantavirus (Old World or New World) responsible, with the exception of the *MV Hondius* outbreak, which is highlighted red for emphasis. Additionally, regions with endemic hantaviruses are colored according to whether the endemic strain is an Old World (pink) or New World (blue) hantavirus. Image created in BioRender.

The global burden associated with hantaviruses reflects two divergent clinical phenotypes. Old World viruses exhibit a case fatality rate (CFR) under 1% in settings with available vaccines and supportive care, whereas New World viruses carry an estimated CFR of 30–60% [17,18]. In 2025, eight countries in the Americas reported 229 cases of hantaviral infections with a CFR of 25.7%. 1,885 cases were reported across Europe in 2023, and thousands of hemorrhagic fever with renal syndrome (HFRS) cases have been reported annually in East Asia [7].

### Molecular virology and host-virus interactions

Hantaviruses (class *Bunyaviricetes,* order *Elliovirales,* family *Hantaviridae*) [19–21] are enveloped, negative-sense, single-stranded RNA viruses with a segmented genome (11–13 Kb) consisting of small (S), medium (M), and large (L) segments, and are associated with an envelope protein containing viral glycoproteins [22,23]. In 2024, the International Committee on Taxonomy of Viruses (ICTV) accepted a proposal to rename the class *Ellioviricetes* to *Bunyaviricetes* in RNA-dependent RNA polymerase (RdRp) phylogeny and to split the order *Bunyavirales* into two orders, a broader classification based on observations of increased clustering into the two distinct orders *Elliovirales* (including families *Hantaviridae* and *Peribunyaviridae*) and *Hareavirales* (including families *Arenaviridae*, *Nairoviridae*, and *Phenuiviridae*) [20,21,24]. Hantaviruses can be dichotomized into two classes based on their geographic distribution: Old World viruses and New World viruses (Fig 1). The key distinction between these classes is the symptoms they cause: Old World viruses cause HFRS, and New World viruses cause hantavirus cardiopulmonary syndrome (HPS or HCPS) [17,25]. ANDV exhibits broad tropism, infecting multiple human cell types, including those of the heart, brain, and lungs, particularly lung epithelial cells. It also induces cellular injury and inflammatory responses while suppressing lipid metabolic pathways in lung epithelial cells [26]. Though less common than pulmonary or cardiac manifestations, New World hantaviruses, particularly ANDV, have also been shown to induce neurological manifestations, such as confusion, meningoencephalitis, pituitary apoplexy, and, albeit rarely, seizures, myelitis, and peripheral neuropathy [27]. Gastrointestinal symptoms, including nausea, vomiting, abdominal pain, and diarrhea, have also been observed in ANDV-infected patients, both preceding and in tandem with the onset of cardiovascular symptoms [28]. However, more investigation is needed to better understand the frequency of onset, the underlying pathophysiological mechanisms, and the clinical significance of these symptoms.

Since the 1950s [29,30], hantaviruses have been identified on every inhabited continent, with pathogenicity varying substantially across strains [22,23]. The differences in pathogenic mechanisms and disease outcomes among viral strains can be partly determined by viral-encoded factors, with several viral entry mechanisms elucidated in recent years. The viral M genome segment encodes glycoprotein precursors that are processed into Gn and Gc subunits, which form tetrameric complexes that help mediate cellular entry [31]. New World hantaviruses utilize protocadherin-1 (PCDH1) to directly engage viral Gn/Gc glycoprotein complexes and facilitate entry into vascular endothelial cells [32,33]. Recent investigations have elucidated the higher-order lattice architecture formed by Gn/Gc heterotetramers on the hantaviral surface, highlighting their role as the primarily necessary and sufficient proteins for viral attachment and entry [34,35]. Analysis of these surface spikes indicates that Gn/Gc complexes may serve as potential targets for antiviral immunotherapeutic interventions, including neutralizing antibodies and structure-guided immunogens [36].

Upon viral entry and genome uncoating in the host-cell cytoplasm, the negative-strand genomes are transcribed into positive-strand mRNA by the viral RdRp. Similar to some other negative-sense RNA viruses, the hantaviral RdRp can also snatch 5’-capped mRNA fragments from host-cell transcripts, allowing the virus to use these sequences as primers for transcription and replication of the viral genome [37]. The nucleocapsid protein engages the viral RdRp and host ribosomes to selectively enhance translation of viral transcripts, while also interfering with protein kinase R (PKR) dimerization and remodeling of host proteostasis pathways, blunting cell-intrinsic antiviral defenses and promoting overall immune evasion [38,39]. Additionally, pathogenic hantaviruses have been shown to dampen early type I interferon (IFN) signaling in infected endothelial cells, thus sensitizing these cells to VEGF-mediated permeability [40,41]. This mechanism may underlie the pathogenesis of HFRS and HCPS. Hantaviruses can evade immune mechanisms by using structural and nonstructural proteins, such as NSs, NP, and Gn, to block RIG-I/MDA5-TBK1 signaling, antagonize IFN induction, and induce TUFM/LC3B-mediated mitophagy to degrade MAVS, respectively [42].

Of particular concern is the capacity of these viruses to evolve, driven by the error-prone RdRP, genetic reassortment events in the L segment, and hypervariable regions in the nucleocapsid protein encoded by the S segment [43,44]. Additionally, the lack of immunity in humans increases the capacity for viral transmission and virulence. This is especially concerning given recent epidemiological trends in Argentina, which suggest that ANDV has a CFR of 34%, even higher than that of strains detected between 2019 and 2025 [45]. Phylogenetic analysis of the ANDV M-segment coding region against reference strains from NCBI revealed that the novel strain associated with the *MV Hondius* outbreak [46] shares 94.26% sequence identity with the ANDV reference sequence (NC_003467.2), indicating evolutionary divergence from the reference strain (Fig 2). Further comparison of this novel M-segment sequence against the NCBI Core Nucleotide Database identified closely related ANDV sequences isolated from human whole blood samples collected in Argentina (south variant) in 2018 (MN258191 and MN258192). These findings suggest that the lineage most closely related to this novel strain may have been circulating in human populations and/or reservoir hosts for nearly eight years, while gradually accumulating mutations over time. Consistent with this inference, the novel strain’s M-coding region shows the highest sequence identity with the 2018 isolates (99.0% and 98.98%, respectively). Notably, the presence of 35 nucleotide mutations but only three amino acid substitutions, two in G1 (T193A and T516I) and one in G2 (A994T), within the glycoprotein-coding regions suggests strong purifying (negative) selection, preserving critical Gn and Gc functions. Overall, these findings indicate that the functional and transmission-relevant properties of ANDV are conserved across strains despite the accumulation of silent (synonymous) mutations, underscoring the importance of sustained genomic surveillance [47].

**Fig 2:**
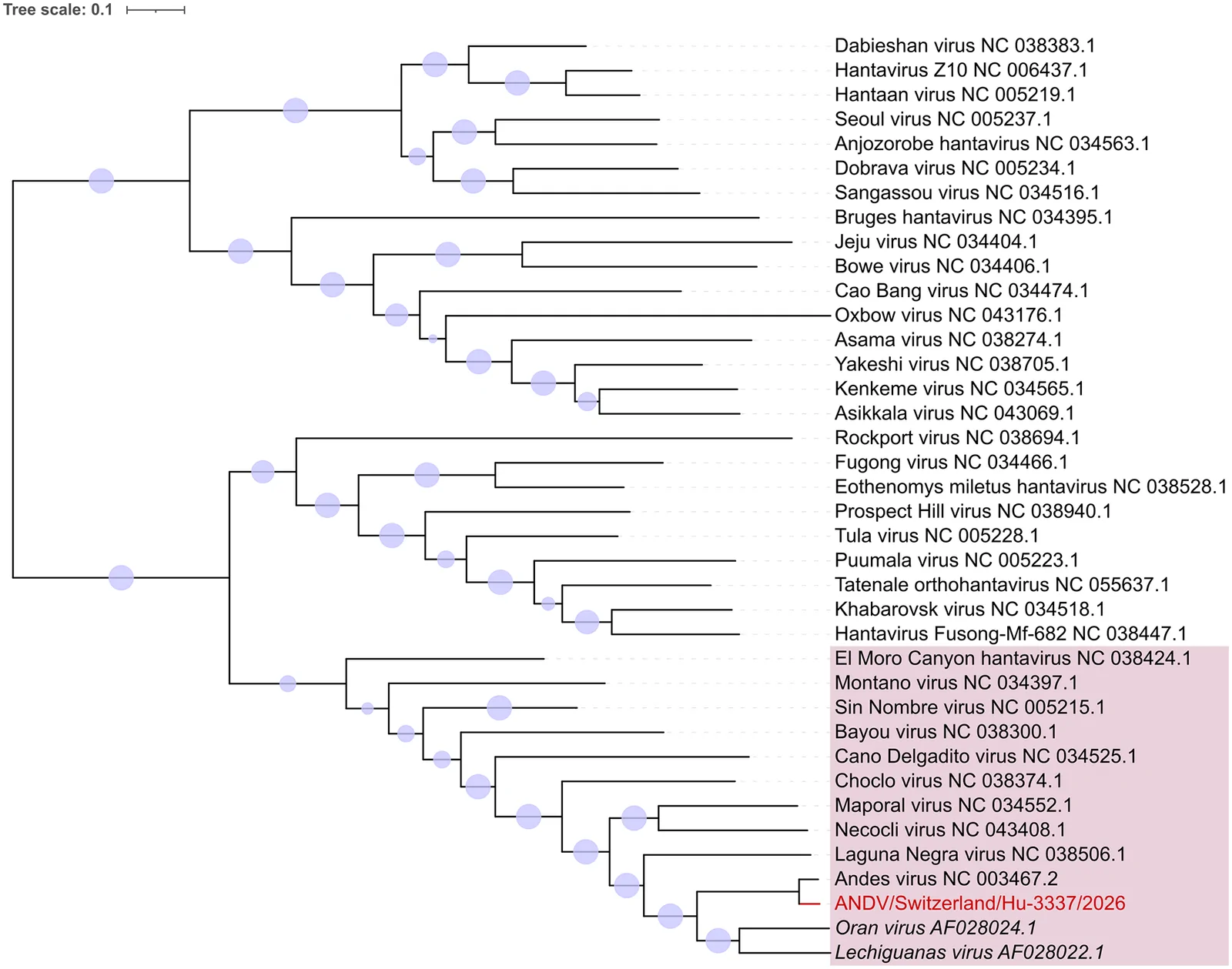
Phylogeny comparing *MV Hondius* ANDV strain to publicly available Hantaviral strains. Maximum-likelihood (ML) phylogenetic tree constructed using 1,000 ultrafast bootstrap replicates (purple circles) for 38 M-segment sequences from the genus *Orthohantavirus*, including 35 species from the National Center for Biotechnology Information (NCBI) RefSeq database, 2 species from GenBank (italicized), and 1 ANDV sequence (red font) obtained from a Swiss resident who had traveled on the *MV Hondius* cruise ship [46]. The clade containing the ANDV strain responsible for the current 2026 outbreak is highlighted in the pink box. Sequences were aligned using MAFFT v7.526, and the resulting alignment was used for ML phylogenetic tree construction in IQ-TREE.

### Human-to-human transmission of ANDV

ANDV is unique in its capacity for human-to-human transmission, first suspected during the 1996 El Bolsón and Bariloche, Argentina, outbreaks and later confirmed at the molecular level soon after [48,49]. The 2018–2019 Epuyén outbreak subsequently provided the most extensive documentation of this transmission route, as it was notable for three distinct superspreader events without any identifiable rodent exposure [50]. Although few additional ANDV outbreaks have been recorded, the *MV Hondius* outbreak provided further epidemiologic observations supporting this transmission route, including secondary cases among close contacts of symptomatic passengers [51]. It should be noted that, given the limited availability of outbreak-associated sequences and lack of continuous temporal sampling, we have avoided inferring direct or continuous viral transmission. Further investigation is needed to better establish the precise mechanisms, viral tropism for upper and lower respiratory tract airway epithelium, infectiousness duration, and full spectrum of transmission routes.

### Clinical management and therapeutics

Management of hantaviral infections primarily consists of supportive care, with early detection and extracorporeal membrane oxygenation (ECMO) in the cardiopulmonary phase remaining the most consistent determinants of survival [52,53]. Although Hantavax, an inactivated bivalent vaccine against HTNV, and a vaccine against SEOV are in active use across China and South Korea, no FDA-approved antivirals or vaccines are currently available for use in Western populations [53]. Several antiviral candidates have been investigated. Vandetanib demonstrated semi-prophylactic efficacy in Syrian hamsters [54], while ribavirin, though promising in animal models, has generally not shown robust efficacy in human clinical trials [55–58]. Additionally, polymerase-targeted antiviral strategies have been investigated in other members of *Bunyaviricites*, with molnupiravir showing inhibitory activity against RdRp of Crimean-Congo hemorrhagic fever virus (order *Hareavirales*, family *Nairoviridae*) [59]. However, further extensive studies are required to develop and evaluate direct-acting antiviral approaches in hantaviruses.

Our team has previously identified potential antiviral candidates, including favipiravir and urolithin B, that demonstrate potent *in vitro* activity against ANDV [26]. Favipiravir, both when used alone and in combination with ribavirin, has been shown to exhibit antiviral activity against Old World HTNV [60]. These compounds were shown to restore cellular metabolism in infected cells, thus warranting further preclinical evaluation and clinical trials. To date, most assessed antiviral candidates have only shown promise *in vitro* or in animal models. It should be noted, however, that one patient from the *MV Hondius* was treated with a combination of intravenous and oral ribavirin, favipiravir, icatibant, and baricitinib, showing complete clinical resolution of viral symptoms after 10 days [61], which highlights the potential importance of combination therapies comprising direct-acting antivirals (e.g., favipiravir) and host-targeted treatments (e.g., icatibant and baricitinib) during late stages of disease.

## Lessons learned and a call to action

The current outbreak has highlighted the need for improved infection prevention, detection, and surveillance infrastructure worldwide. Our current public health infrastructure can be better optimized to respond rapidly to outbreaks of neglected zoonotic pathogens, such as hantaviruses. As ANDV can spread through contact with infected rodents or their urine, saliva, or feces, surveillance and early ecological warning must be prioritized, including regular monitoring of rodent reservoir populations in high-incidence areas. It has been demonstrated that a synchronous, rapid increase in rodent populations in northwestern Argentina predictably preceded an HPS outbreak in 2023 [16,62]. As climate change continues to alter the geographic range and abundance of vector-borne organisms, the risk posed to global populations by hantaviruses could expand substantially. The current leading hypothesis for the origin of the cruise ship outbreak, according to Argentine public health officials, is that patient zero, who was birdwatching in Ushuaia, a small town in the Tierra del Fuego region of Southern Argentina, was infected by exposure to rodents from the landfill, though this has yet to be confirmed categorically at the time of writing [63]. Given its status as a region well-known for its biodiversity and for attracting international birdwatchers and wildlife enthusiasts, and its having served as the starting point for prior and current ANDV outbreaks, there is a crucial need for active and consistent disease surveillance in Tierra del Fuego and Patagonia. These gaps echo the broader concerns regarding the limits of infectious disease preparedness in close-quarter travel settings and constrained on-board diagnostic capacity [64], as well as the impact of early public health reporting on multi-agency coordination efforts [65].

While the initial multiplex respiratory pathogen panel used to screen the patients lacked sufficient sensitivity to detect ANDV infection, the establishment of molecular diagnostics (e.g., PCR), serologic assays (IgG and IgM), next-generation sequencing (NGS), whole-genome sequencing (WGS), and metagenomic sequencing assays for this nonselect agent virus is urgently needed in public health laboratories to strengthen preparedness for potential future outbreaks and pandemics worldwide. During the *MV Hondius* outbreak, the CDC Health Alert Network (HAN) notified clinicians and health departments about a new cluster of ANDV infections and provided recommendations for US public health departments, clinical laboratories, and healthcare workers regarding case identification, diagnostic testing, and biosafety considerations in clinical laboratories. Although CLIA-certified assays for detecting New World hantavirus IgM and IgG antibodies are currently available to the CDC, select state public health laboratories, and Quest Diagnostics [66], broader implementation of these assays across public health laboratories would further enhance preparedness and response capacity. Additionally, given the ever-evolving nature of these viral genomes, these assays will need to be updated regularly to detect new strains. Epidemiologic investigations and infection prevention measures, including close monitoring of ill persons, are essential for quickly mitigating or preventing future viral transmission. Although the overall public health risk posed by ANDV is low compared to that of SARS-CoV-2 or the influenza virus, individuals who have direct physical contact with or prolonged exposure in close or enclosed settings to symptomatic persons with confirmed or suspected ANDV infection may face an elevated risk of transmission, particularly through contact with contaminated body fluids or fomites [66]. Notably, large international sports or other events, such as the FIFA Women’s World Cup or the 2028 Olympics, and larger gatherings could plausibly increase opportunities to introduce or transmit emerging and re-emerging pathogens of public health concern. Although this possibility remains hypothetical, the unprecedented movement of international travelers underscores the importance of continued surveillance and preparedness, even though this risk, while likely low, has not yet been quantified specifically for ANDV. Therefore, rigorous public health preparedness and preventive measures are warranted, including genomic surveillance of pandemic-potential pathogens through amplicon-based, hybrid-capture, and/or agnostic metagenomic analyses of wastewater. Such monitoring systems can facilitate early detection and provide timely warning signals of emerging threats.

Research infrastructure must be dramatically expanded. Hantavirus research requires biosafety level (BSL)-3 facilities for viral culture and BSL-4 for animal experimentation, which pose severe limitations to most academic researchers due to the extensive cost of labor, equipment, infrastructure, and certification to sustain these facilities [67]. Laboratory transmission of hantaviruses via aerosol routes has been extensively documented [68–71], thus requiring high-containment protocols. However, this could hamper access to conducting hantavirus research and limit cross-institutional collaboration, especially across countries.

Additionally, the immunopathogenic mechanisms of hantavirus infection are yet to be fully elucidated. HCPS has been shown to be driven by immune-mediated capillary leak, with CD8+ T cells and proinflammatory cytokines influencing disease severity [72]. However, it is unknown why young, immunocompetent individuals are disproportionately affected compared to other viruses, and what triggers the shift in the immune system from protective to pathological responses. Given the GI symptoms observed in infected individuals in this outbreak, there is a need to more rigorously evaluate whether ANDV can replicate in the GI tract. Atypical presentations of hantaviral infection, such as mimicking acute viral hepatitis and neurological symptoms, are an underrecognized but important consideration that could complicate timely recognition of hantaviral infection [73].

### Proposed areas for intervention

This latest ANDV outbreak exemplifies the risk that neglected zoonotic pathogens pose to global health and highlights the importance of sustained preparedness efforts. Given the high lethality, uncertain pandemic-potential, complex geographic distribution, and ecologically driven transmission dynamics of ANDV, immediate areas of intervention include expanding CLIA-certified ANDV serologic and molecular testing capacity beyond the current reference laboratories. Additionally, contact tracing and biosafety protocols in affected regions should be evaluated for optimization given longer incubation periods after viral exposure. Another important area is establishing regular rodent reservoir surveillance in the Tierra del Fuego and Patagonia regions, as well as standardizing diagnostic protocols across public health laboratories. In the long term, there is a need to continue clinical translation of promising therapeutic and prophylactic candidates. Several vaccine candidates, including virus-like particle, inactivated, recombinant, viral vector, and nucleic acid types, are in various stages of development or undergoing early-stage clinical trials against HTNV, PUUV, SEOV, ANDV, and DOBV [52,74]. In addition, establishing robust pandemic preparedness infrastructure that integrates cross-cutting genomic epidemiology, climate-linked surveillance, and prophylactic and therapeutic development pipelines will be critical to strengthening future outbreak response and resilience.
